# Long-term efficacy and safety of pegunigalsidase alfa administered every 4 weeks in adults with Fabry disease: results from up to 5 years of the BRIGHT F51 phase III, open-label extension study

**DOI:** 10.1186/s13023-026-04303-8

**Published:** 2026-03-20

**Authors:** Myrl Holida, Aleš Linhart, Nicola Longo, Eric Wallace, Camilla Tøndel, Derralynn Hughes, David G. Warnock, Antonio Pisani, François Eyskens, Patrick Deegan, Ulla Feldt-Rasmussen, Ozlem Goker-Alpan, Ankit Mehta, Giovanni Piotti, Vito Fichera, Meng Wang, Raul Chertkoff, Stephen Waldek, William R. Wilcox, John A. Bernat

**Affiliations:** 1 Division of Medical Genetics and Genomics, Stead Family Department of Pediatrics, University of Iowa Health Care, Iowa City, IA USA; 2 Charles University, General University Hospital, Prague, Czech Republic; 3 Division of Clinical Genetics, Department of Human Genetics, University of Los Angeles California, Los Angeles, CA USA; 4 Division of Nephrology, Department of Medicine, University of Alabama at Birmingham, Birmingham, AL USA; 5 Department of Clinical Science, University of Bergen and Department of Pediatrics, Haukeland University Hospital, Bergen, Norway; 6 Lysosomal Storage Disorders Unit, Royal Free London NHS Foundation Trust and University College London, London, UK; 7 Department of Public Health, University Federico II of Naples, Naples, Italy; 8 Antwerp University Hospital UZA, Edegem, Belgium; 9 Lysosomal Disorders Unit, Cambridge University Hospitals NHS Foundation Trust and University of Cambridge, Cambridge, UK; 10 Department of Nephrology and Endocrinology and Department of Growth and Reproduction, Rigshospitalet and Faculty of Health and Clinical Sciences, Copenhagen University, Copenhagen, Denmark; 11 Lysosomal and Rare Disorders Research and Treatment Center, Fairfax, VA USA; 12 Baylor University Medical Center, Dallas, TX USA; 13 Chiesi Farmaceutici S.p.A, Parma, Italy; 14 Department of Product Development, Protalix Biotherapeutics, Carmiel, Israel; 15 University of Sunderland, Sunderland, UK; 16 Department of Human Genetics, Emory University School of Medicine, Atlanta, GA USA

**Keywords:** Lysosomal storage disorders, Fabry disease, Enzyme replacement therapy, Pegunigalsidase alfa, eGFR, Open-label extension

## Abstract

**Background:**

Enzyme replacement therapies (ERTs) approved for Fabry disease require infusions every 2 weeks (E2W). Pegunigalsidase alfa, a PEGylated ERT with a prolonged half-life vs. other ERTs, may allow extension of the dosing interval to every 4 weeks (E4W).

BRIGHT F51 (NCT03614234) is an ongoing phase III, open-label extension study evaluating long-term efficacy and safety of pegunigalsidase alfa 2 mg/kg E4W in adults with Fabry disease previously treated with agalsidase alfa or beta E2W for ≥ 3 years who completed one year of pegunigalsidase alfa treatment in the BRIGHT study. This interim analysis reports results following 3–5 years of treatment (cutoff date December 31, 2022).

**Results:**

Twenty-nine patients were enrolled. Median (interquartile range [IQR]) annualized eGFR slope during treatment was ‒2.2 (‒2.9; ‒1.1) mL/min/1.73 m^2^/year (males: ‒2.4 [‒2.9; ‒1.0, *n = *23]; females: ‒1.8 [‒2.4; ‒1.3, *n *= 6]; anti-drug antibody [ADA]-positive: ‒2.6 [‒4.0; ‒1.7, *n =* 9 all male]; ADA-negative: ‒1.8 [‒2.7; ‒0.6, *n = *20]). Median (IQR) change in plasma lyso-Gb3 from baseline to Week 208 was 3.2 (‒3.9; 8.5, *n = *17) nM in males; concentrations remained low and stable in females. Overall, 51/477 treatment-emergent adverse events in 13 patients (45%) were considered treatment-related (all mild/moderate). Nine patients (31%) experienced mild/moderate infusion-related reactions. One patient developed transient *de novo *ADAs.

**Conclusions:**

Long-term treatment with pegunigalsidase alfa 2 mg/kg E4W was well-tolerated and maintained disease stability, especially in females and ADA-negative males; more data are needed to better understand outcomes in ADA-positive males. Clinical outcomes should be closely monitored during E4W treatment. The final results of this extension study will further assess the feasibility of this dosing regimen.

**Trial registration details:**

ClinicalTrials.gov, NCT03614234. Registered July 30, 2018; https://clinicaltrials.gov/study/NCT03614234.

**Graphical Abstract:**

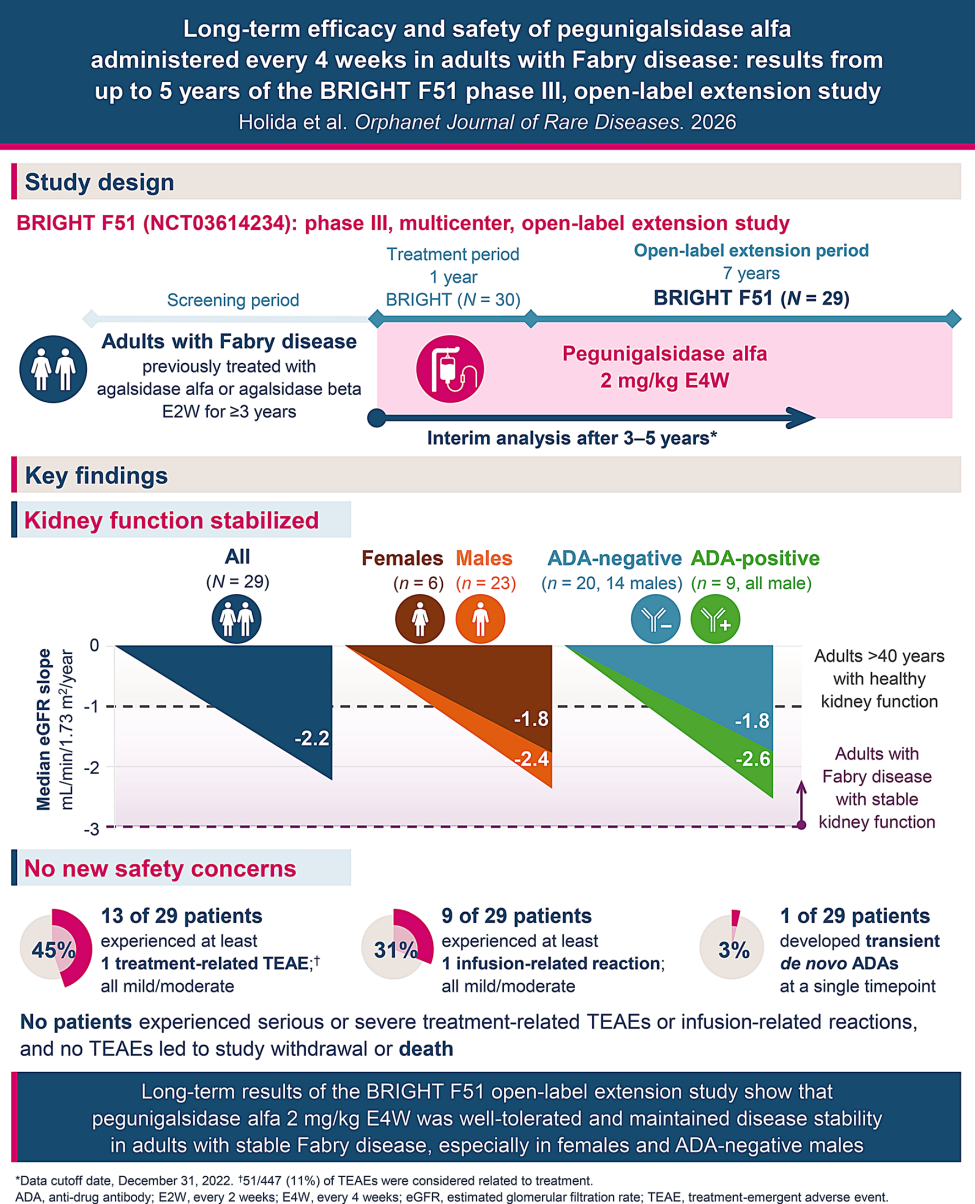

**Supplementary Information:**

The online version contains supplementary material available at 10.1186/s13023-026-04303-8.

## Introduction

Fabry disease (OMIM #301500) is a rare X-linked disorder caused by pathogenic variants in the galactosidase alpha (*GLA*) gene [1–3]. These variants result in deficiency of the lysosomal enzyme alpha-galactosidase A (α-Gal A) and accumulation of globotriaosylceramide (Gb3) and globotriaosylsphingosine (lyso-Gb3) [1–3]. The buildup of these sphingolipids may affect multiple organ systems, ultimately leading to life-threatening complications [1–3].

Treatment options for Fabry disease include enzyme replacement therapy (ERT) with recombinant α-Gal A and pharmacological chaperone therapy [4–8]. Agalsidase alfa and agalsidase beta are ERTs administered intravenously every 2 weeks (E2W) at 0.2 mg/kg and 1 mg/kg, respectively [9, 10]. These can improve clinical outcomes and quality of life (QoL) for patients [4–7, 11, 12]. Disadvantages include the need for infusions E2W and risk of infusion-related reactions and anti-drug antibodies (ADAs) [4, 9, 10, 13, 14]. While the oral chaperone, migalastat, may address some limitations of ERTs, it is only suitable for those with amenable *GLA *mutations [4, 8, 15, 16].

Pegunigalsidase alfa 1 mg/kg E2W is approved for the treatment of Fabry disease [17, 18]. It is a recombinant human α-Gal A covalently conjugated with polyethylene glycol (PEG) [19]. Due to PEGylation, pegunigalsidase alfa demonstrated enhanced enzymatic stability, prolonged circulatory half-life, and improved tissue distribution while maintaining enzyme activity, relative to agalsidase alfa and beta in pre-clinical studies [19]. The extended circulatory half-life of pegunigalsidase alfa (~80 h) vs. other ERTs (≤ 2 h) [9, 10] was confirmed in clinical studies [17, 20]. PEGylation may also decrease the affinity of pre-existing ADAs to pegunigalsidase alfa by masking antigenic epitopes, thereby reducing ADA-mediated inhibition of the enzyme activity [21]. Although ERTs carry a risk of hypersensitivity reactions, clinical data suggest pegunigalsidase alfa may be associated with lower rates of such events. In a study involving ERT-naïve patients, only a few cases of ADAs, mostly transient, were observed [22]. Across the clinical development program—including the pivotal BRIDGE (ClinicalTrials.gov identifier: NCT03018730) [23] and BALANCE (NCT02795676) [24] trials—pegunigalsidase alfa was generally well-tolerated and demonstrated improvements in biomarker and clinical outcomes in both ERT-naïve patients and those switching from other ERTs. In total, over 140 patients were evaluated, with 111 receiving pegunigalsidase alfa 1 mg/kg E2W [17, 18, 20, 22–25].

Extending pegunigalsidase alfa infusion intervals from E2W to every 4 weeks (E4W) is supported by its greater stability and prolonged half-life compared with other ERTs [19, 20]. This extended dosing schedule is approved in the European Union and is currently under investigation in other regions for patients with Fabry disease [26]. Decreasing dosing frequency has shown to improve patient adherence and satisfaction, indicating reduced therapeutic burden [27, 28]. Pegunigalsidase alfa 2 mg/kg E4W has been evaluated in BRIGHT (NCT03180840), an open-label, switchover study that assessed the pharmacokinetics, safety, and efficacy of this dosing regimen over 12 months in adults previously treated with agalsidase alfa or beta [25]. In this study, pegunigalsidase alfa E4W showed no new safety signals nor *de novo *ADA development, while maintaining disease stability in most patients [25]. Average pegunigalsidase alfa plasma concentrations following each 4-week infusion interval were substantially above the lower limit of quantification, confirming its extended circulatory availability [25].

Here, we report interim efficacy and safety results of the ongoing open-label extension (OLE) of the BRIGHT study (NCT03614234) following 3–5 years of treatment with pegunigalsidase alfa 2 mg/kg E4W. A plain language summary of this article is available as Additional file 1.

## Methods

### Study design

This interim analysis assessed long-term efficacy and safety of pegunigalsidase alfa 2 mg/kg E4W in adult patients with Fabry disease who completed the phase III BRIGHT study [25]. After entry into the extension study, patients continued to receive pegunigalsidase alfa at the same dosing regimen and infusion duration as upon completion of BRIGHT [25], with the same premedication (if used previously) and in the same setting. Home infusions were permitted depending on local regulations and if the investigator and medical monitor agreed these were safe. For any cases of recognized clinical deterioration, the protocol permitted treatment modification to pegunigalsidase alfa 1 mg/kg E2W at the investigator’s discretion.

The ongoing OLE was designed for patients to receive pegunigalsidase alfa 2 mg/kg E4W (± 3 days) for up to 92 infusions over 84 months (7 years), after completion of 12 months (1 year) of treatment in the primary BRIGHT study (total treatment period of 8 years). The baseline of the OLE was defined as the assessments made at the baseline visit of the primary BRIGHT study; if these were not available, the baseline corresponded to the last assessment before receiving the first dose of pegunigalsidase alfa in BRIGHT. The screening visit of the OLE took place at the last infusion visit in BRIGHT at Week 52 (1 year). The cutoff date for this interim analysis was December 31, 2022.

The study was conducted in accordance with the Declaration of Helsinki and Good Clinical Practice guidelines at 14 study sites in Belgium, Czech Republic, Denmark, Italy, Norway, the United Kingdom, and the United States of America. Patients provided written informed consent, and the study protocol and any amendments were approved by an independent ethics committee or institutional review board.

### Patients

To enroll in the OLE, patients must have completed the primary BRIGHT study, for which the key participation criteria were previous treatment with agalsidase alfa or beta E2W for ≥ 3 years, estimated glomerular filtration rate (eGFR) at screening ≥ 30 mL/min/1.73 m^2^, and annualized linear eGFR slope at screening less negative than ‒2 mL/min/1.73 m^2^/year [25]. Detailed inclusion and exclusion criteria for BRIGHT have previously been published [25].

### Endpoints

Key efficacy endpoints included change in eGFR, calculated using the Chronic Kidney Disease – Epidemiology Collaboration (CKD-EPI) equation based on serum creatinine values [29]; annualized eGFR slope, calculated for each patient based on baseline and all available post-baseline eGFR values using linear regression; and change in plasma lyso-Gb3 concentrations, as per the analytical assays and methods described in the BRIGHT study [25]. Urine protein to creatinine ratio (UPCR) was determined by a spot urine test and categorized according to the Kidney Disease: Improving Global Outcomes (KDIGO) guidelines (normal to mildly increased < 0.15 g/g, 0.15 g/g ≤ moderately increased ≤ 0.5 g/g, and severely increased > 0.5 g/g) [30]. Change in disease severity was evaluated by the Mainz Severity Score Index (MSSI) clinical subdomains (cardiovascular [score range 0–20], neurological [0–20], renal [0–18], and general [0–18]; for all subdomains, higher scores indicate increased severity) and overall score (sum of all subdomains; mild < 20, 20 ≤ moderate ≤ 40, and severe > 40) [31, 32]. Patient-reported outcomes included change in pain, evaluated using the short-form Brief Pain Inventory (BPI) [33], and change in QoL, assessed using the EuroQol 5-Dimensions 5-Levels Questionnaire (EQ-5D-5L) [34].

The key safety endpoint was the evaluation of treatment-emergent adverse events (TEAEs, assessed by Common Terminology Criteria for Adverse Events [CTCAE v4.03] [35]), including severe, serious, and treatment-related TEAEs, as well as TEAEs leading to study withdrawal or death. Other safety endpoints included extent of exposure, duration of infusion, incidence of infusion-related reactions (defined as definitely, probably, or possibly treatment-related TEAEs occurring during the infusion or within 2 h after its completion), and ADA status post-treatment. Details on bioanalytical methods of ADA and neutralizing antibody (nAb) assessments have previously been published [25, 36].

Efficacy and safety endpoints were assessed at baseline, screening (Week 52), and approximately every 6 months at the OLE evaluation visits (Weeks 80, 108, 132, 160, 184, 208, 232, and 256). Exceptions were MSSI scores, which were recorded at yearly intervals (baseline; screening; and Weeks 108, 160, 208, and 256), and TEAEs, which were evaluated throughout the study.

### Statistical analysis

The study was descriptive in nature. Descriptive statistics are shown for continuous variables, including arithmetic mean, standard deviation (SD), median, interquartile range (IQR), and range. For categorical variables, frequency counts and percentages are provided. Rates per 100 patient-years are presented for AEs, and rates adjusted per 100 infusions are given for infusion-related reactions.

The study populations included the efficacy set and safety set (based on all patients who received a dose [partial or complete] of pegunigalsidase alfa in the OLE). The efficacy analyses included data collected from patients while they were receiving pegunigalsidase alfa 2 mg/kg E4W only (i.e., data collected from patients after they switched to pegunigalsidase alfa 1 mg/kg E2W were not included in these analyses). Additional safety analyses for extent of exposure to pegunigalsidase alfa, duration of infusion, and infusion-related reactions included data exclusively on the 2 mg/kg E4W dosing regimen as well. Summarized data were integrated from the primary BRIGHT study to the cutoff date of the OLE in a longitudinal manner.

Subgroup analyses included stratification according to sex (male and female), since levels of lyso-Gb3 differ between men and women with Fabry disease [37–39]. Other subgroup analyses were conducted according to baseline ADA status (positive vs. negative) and eGFR values (> 120 vs. ≤ 120 mL/min/1.73 m^2^ [including 90 < eGFR ≤ 120, 60 < eGFR ≤ 90, and 30 < eGFR ≤ 60]). Due to the small number of patients with severe proteinuria (UPCR > 0.5 g/g), subgroup analyses for UPCR were not performed.

## Results

### Patients

**Fig. 1:**
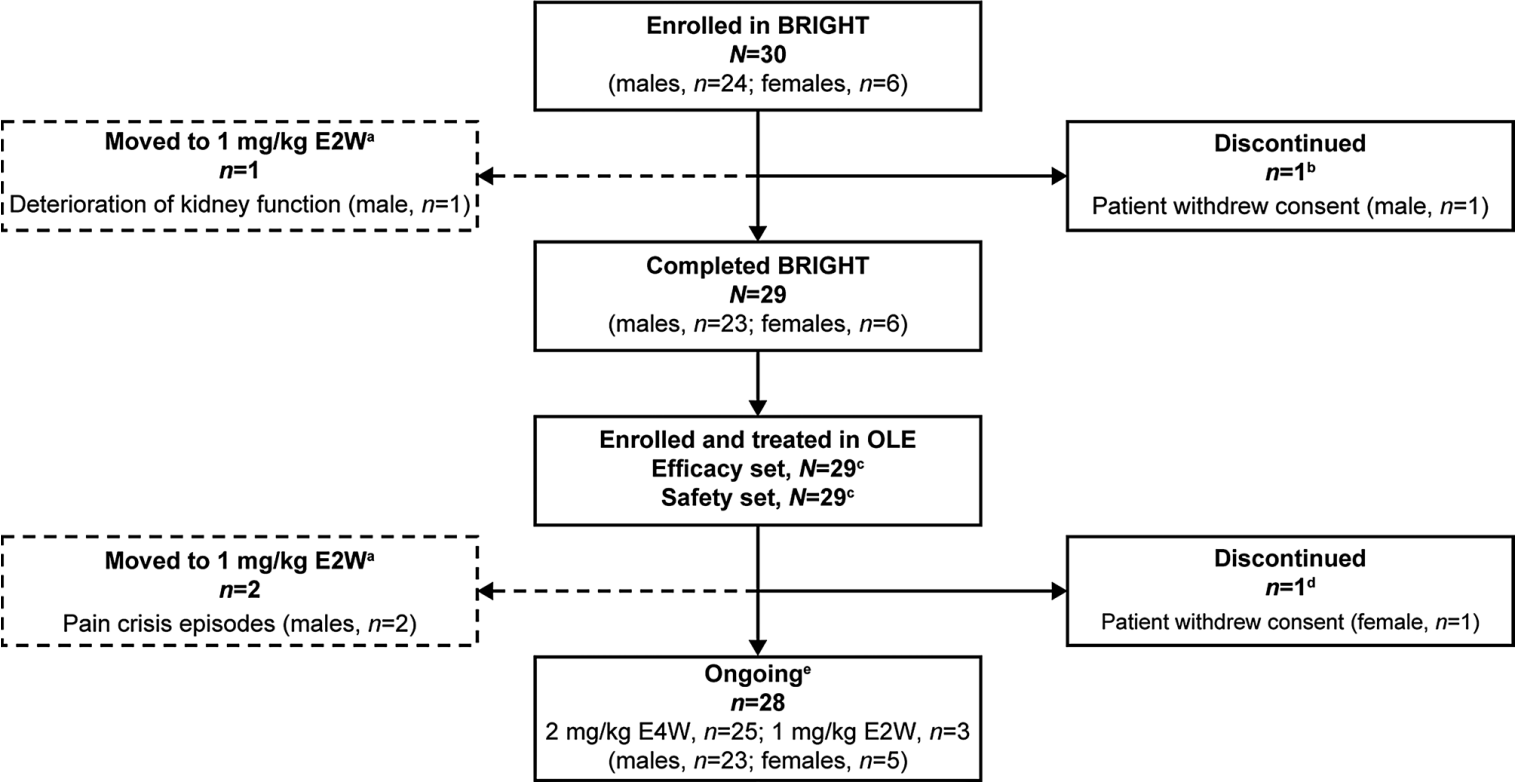
Patient disposition. ^a^Patients whose pegunigalsidase alfa administration regimen was modified from 2 mg/kg E4W to 1 mg/kg E2W continued study participation after the modification. ^b^One male patient who received the first infusion of pegunigalsidase alfa 2 mg/kg at baseline withdrew consent after this visit due to a traffic accident unrelated to Fabry disease. ^c^The efficacy and safety sets included all 29 patients treated in the OLE. The efficacy analyses and safety analyses for infusion-related reactions included data collected from patients while they were receiving pegunigalsidase alfa 2 mg/kg E4W only. ^d^One female patient withdrew consent and discontinued study participation during the OLE after ~ 3 years of treatment with pegunigalsidase alfa 2 mg/kg E4W. The patient reported tiredness from the 3rd week after the first infusion. ^e^At data cutoff: December 31, 2022. E2W, every 2 weeks; E4W, every 4 weeks; OLE, open-label extension

A total of 29 patients (23 [79.3%] male and 6 [20.7%] female; Fig. 1) completed the primary BRIGHT study and were enrolled in the ongoing OLE. One female patient withdrew consent and discontinued OLE participation due to tiredness (Fig. 1). At the time of this interim analysis, 28 patients were continuing participation and had completed a total of at least 36 months (3 years) of treatment with pegunigalsidase alfa (i.e., 12 months in BRIGHT and at least 24 months in the current OLE study). As patients were enrolled at different timepoints in the OLE, 25 patients (86.2%) had completed a total of 48 months (4 years), and 4 patients (13.8%) a total of 60 months (5 years) of treatment at the data cutoff.

The pegunigalsidase alfa dosing regimen was modified to 1 mg/kg E2W for one patient during the primary BRIGHT study and for two patients during the OLE; the patients (all male) continued study participation post-modification (Fig. 1). The first patient’s regimen was modified at Week 40 due to deterioration of kidney function (from an eGFR of 30.3 mL/min/1.73 m^2^ at baseline to an eGFR of 24.0 mL/min/1.73 m^2^ at Week 40). This patient had pre-existing immunoglobulin G antibodies at baseline and high titers of cross-reacting anti-pegunigalsidase alfa antibodies and nAbs at all timepoints prior to treatment modification. The other two patients’ regimens were modified at Weeks 84 and 220, respectively, due to pain crisis episodes. The first of these two patients experienced 18 TEAEs indicative of pain over approximately 19.5 months while receiving pegunigalsidase alfa E4W (including one severe event of generalized pain and 6 infusion-related reactions) and 2 pain episodes while receiving infusions E2W over approximately 38 months (one infusion-related reaction). This patient had neutralizing ADAs at all timepoints preceding the dosing modification. The second patient experienced 6 TEAEs indicative of pain over approximately 51 months on the E4W administration regimen (including one severe fever pain crisis) and one pain episode during the approximate 5 months after moving to the E2W dosing schedule. None of these events were considered treatment-related. This patient was ADA-positive at baseline, but seroreverted at Week 24 of the primary BRIGHT study.

Patient demographics and baseline characteristics (overall and stratified by sex and ADA status) are presented in Table 1.

**Table 1:** Patient demographics and baseline characteristics (safety population)

Characteristic	Male (*n* = 23)	Female (*n* = 6)	ADA-negative (*n* = 20)	ADA-positive (*n* = 9)	Overall (*N* = 29)
**Age, years**					
Mean (SD) Median (range)	39.8 (12.2) 40.0 (19; 58)	45.2 (5.3) 46.5 (37; 52)	44.1 (10.5) 46.5 (19; 58)	33.8 (10.0) 35.0 (20; 48)	40.9 (11.3) 41.0 (19; 58)
**Age at ERT start, years**					
Mean (SD) Median (range)	30.2 (13.8) 31.0 (7; 51)	38.5 (4.0) 38.5 (33; 45)	36.1 (11.5) 36.5 (7; 51)	22.7 (10.9) 19.0 (10; 41)	31.9 (12.8) 35.0 (7; 51)
**Previous ERT, *n* (%)**					
Agalsidase alfa Agalsidase beta	5 (21.7) 18 (78.3)	2 (33.3) 4 (66.7)	7 (35.0) 13 (65.0)	0 (0.0) 9 (100.0)	7 (24.1) 22 (75.9)
**Duration of previous ERT, years**					
Mean (SD) Median (range)	9.3 (5.0) 8.8 (2.2; 18.4)	5.7 (2.9) 5.2 (1.3; 9.4)	7.8 (4.7) 5.7 (1.3; 16.9)	10.1 (5.1) 11.5 (2.8; 18.4)	8.5 (4.9) 6.9 (1.3; 18.4)
**eGFR, mL/min/1.73 m**^**2a,b**^					
* n* Mean (SD) Median (IQR) Range	23 100.7 (23.8) 102.3 (86.4; 119.5) 30.3; 135.9	6 94.7 (16.6) 100.4 (95.9; 103.7) 61.7; 106.1	20 96.2 (17.3) 97.8 (86.3; 106.1) 58.5; 135.9	9 106.7 (30.8) 119.5 (102.3; 123.5) 30.3; 132.2	29 99.4 (22.3) 102.1 (91.6; 110.0) 30.3; 135.9
**Annualized eGFR slope, mL/min/1.73 m**^**2**^**/year**^**c**^					
* n* Mean (SD) Median (IQR) Range	23 ‒1.1 (3.2) ‒0.6 (‒2.6; 0.4) ‒10.5; 3.6	6 ‒4.3 (4.7) ‒3.1 (‒3.7; ‒1.4) ‒13.6; ‒0.7	20 ‒2.3 (4.2) ‒1.7 (‒3.4; 0.2) ‒13.6; 3.6	9 ‒0.6 (2.1) ‒0.6 (‒1.7; 0.1) ‒4.3; 3.3	29 ‒1.8 (3.7) ‒1.1 (‒3.1; 0.1) ‒13.6; 3.6
**Kidney function: eGFR > 120 mL/min/1.73 m**^**2**^					
Yes, *n *(%) No, *n *(%)	5 (21.7) 18 (78.3)	0 (0.0) 6 (100.0)	1 (5.0) 19 (95.0)	4 (44.4) 5 (55.6)	5 (17.2) 24 (82.8)
**Plasma lyso-Gb3, nM**					
* n* Mean (SD) Median (IQR) Range	23 23.3 (18.3) 17.2 (12.1; 32.8) 0.5; 75.1	6 4.4 (2.5) 4.4 (2.9; 5.9) 0.7; 7.8	20 12.3 (10.8) 9.3 (4.7; 16.0) 0.5; 39.0	9 35.1 (21.7) 29.0 (18.2; 50.5) 13.8; 75.1	29 19.4 (18.1) 14.5 (6.2; 23.3) 0.5; 75.1
**Presence of severe proteinuria**^**d**^					
Yes, *n *(%) No, *n *(%)	2 (8.7) 21 (91.3)	0 (0.0) 6 (100.0)	0 (0.0) 20 (100.0)	2 (22.2) 7 (77.8)	2 (6.9) 27 (93.1)
**Treatment with ACEi or ARB**					
Yes, *n *(%) No, *n *(%)	9 (39.1) 14 (60.9)	1 (16.7) 5 (83.3)	7 (35.0) 13 (65.0)	3 (33.3) 6 (66.7)	10 (34.5) 19 (65.5)
**Use of pre-medication for ERT infusion prior to enrollment**^**e**^					
Yes, *n *(%) No, *n *(%)	7 (30.4) 16 (69.6)	2 (33.3) 4 (66.7)	5 (25.0) 15 (75.0)	4 (44.4) 5 (55.6)	9 (31.0) 20 (69.0)
**ADA status for pegunigalsidase alfa**^**f**^					
Positive, *n *(%) Negative, *n *(%)	9 (39.1) 14 (60.9)	0 (0.0) 6 (100.0)	0 (0.0) 20 (100.0)	9 (100.0) 0 (0.0)	9 (31.0) 20 (69.0)
**ADA status for agalsidase alfa**^**f**^					
* n* Positive, *n *(%) Negative, *n *(%)	5 0 (0.0) 5 (100.0)	2 0 (0.0) 2 (100.0)	7 0 (0.0) 7 (100.0)	0 0 (0.0) 0 (0.0)	7 0 (0.0) 7 (100.0)
**ADA status for agalsidase beta**^**f**^					
* n* Positive, *n *(%) Negative, *n *(%)	18 10 (55.6) 8 (44.4)	4 0 (0.0) 4 (100.0)	13 1 (7.7) 12 (92.3)	9 9 (100.0) 0 (0.0)	22 10 (45.5) 12 (54.5)

*Note: *All patients enrolled in the study were White. ^a^Estimated using the CKD-EPI equation. ^b^The higher mean and median values of eGFR in males vs. females were due to all patients with eGFR > 120 mL/min/1.73 m^2^ being male. Similarly, the higher mean and median values of eGFR in ADA-positive vs. ADA-negative patients were due to a higher proportion of patients with eGFR > 120 mL/min/1.73 m^2^ in this subgroup. ^c^Annualized eGFR slope at baseline was based on historical, screening, and baseline values of eGFR. ^d^Severe proteinuria defined as a UPCR > 0.5 g/g according to the KDIGO classification. ^e^Infusion premedications were identified based on the classification on the concomitant medication eCRF. ^f^ADA status for pegunigalsidase alfa based on the results of the IgG for pegunigalsidase alfa at baseline; ADA status for agalsidase alfa based on the results of the IgG for agalsidase alfa at baseline among patients who were treated with agalsidase alfa prior to the switch; ADA status for agalsidase beta based on the results of the IgG for agalsidase beta at baseline among patients who were treated with agalsidase beta prior to the switch.

Abbreviations: ACEi, angiotensin-converting enzyme inhibitor; ADA, anti-drug antibody; ARB, angiotensin receptor blocker; CKD-EPI, Chronic Kidney Disease – Epidemiology Collaboration; eCRF, electronic case report form; eGFR, estimated glomerular filtration rate; ERT, enzyme replacement therapy; IgG, immunoglobulin G; IQR, interquartile range; KDIGO, Kidney Disease: Improving Global Outcomes; lyso-Gb3, globotriaosylsphingosine; SD, standard deviation; UPCR, urine protein to creatinine ratio

Mean (SD) age at enrollment was 40.9 (11.3) years (range: 19; 58), and the mean (SD) duration of previous ERT was 8.5 (4.9) years. A total of 22 (75.9%) patients (18 males and 4 females) had been previously treated with agalsidase beta; the remaining 7 (24.1%) patients (5 males and 2 females) had been previously treated with agalsidase alfa. In total, 5 (17.2%) patients (all male, 4/5 ADA-positive) presented with eGFR values > 120 mL/min/1.73 m^2^ at study entry. Severe proteinuria was noted in 2 (6.9%) patients (both male). A comprehensive overview of pathogenic *GLA *variants in the study population is presented in Table 2.

**Table 2:** Pathogenic *GLA *variants in the study population

Patient No.	Sex	*GLA ***variant** (NM_000169.3)	Predicted α-Gal A protein change
1	Male	c.830G>A	p.Trp277*
2	Male	c.679C>T	p.Arg227*
3	Male	c.95T>C	p.Leu32Pro
4	Female	c.677G>A	p.Trp226*
5	Male	c.680G>A	p.Arg227Gln
6	Male	c.644A>G	p.Asn215Ser
7	Male	c.679C>T	p.Arg227*
8	Male	c.142G>C	p.Glu48Gln
9	Male	c.(194+1_195-1)_(369+1_370-1)del	p.Ser65Argfs*7
10	Male	c.85dup	p.Ala29Glyfs*2
11	Male	c.779G>A	p.Gly260Glu
12	Male	c.679C>T	p.Arg227*
13	Male	c.155G>C	p.Cys52Ser
14	Male	c.568del	p.Ala190Profs*2
15	Male	c.281G>A	p.Cys94Tyr
16	Female	c.1025G>A	p.Arg342Gln
17	Male	c.124A>G	p.Met42Val
18	Female	c.966C>A	p.Asp322Glu
19	Male	c.1212_1214del	p.Arg404del
20	Male	c.427G>A	p.Ala143Thr
21	Female	c.427G>A	p.Ala143Thr
22	Female	c.277G>A	p.Asp93Asn
23	Male	c.801+48T>G	p.Leu268Valfs*4
24	Male	c.801+48T>G	p.Leu268Valfs*4
25	Male	c.679C>T	p.Arg227*
26	Male	c.334C>T	p.Arg112Cys
27	Female	c.680G>C	p.Arg227Pro
28	Male	c.863C>A	p.Ala288Asp
29	Male	c.901C>G	p.Arg301Gly

*Note: *All patients had a clinically documented diagnosis of Fabry disease as per the inclusion criteria of the primary BRIGHT study [25]

### Efficacy

#### Renal function: absolute change in eGFR

**Fig. 2:**
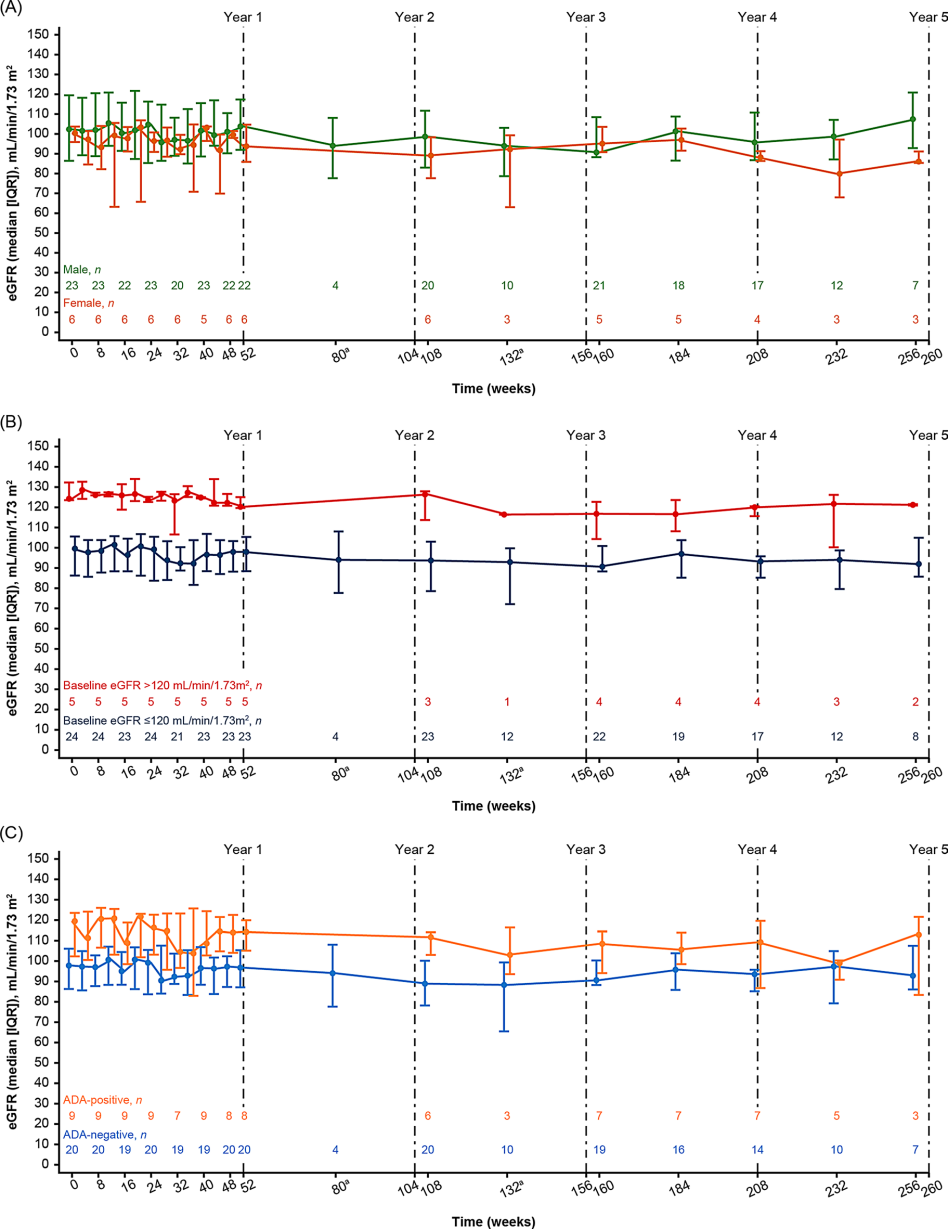
eGFR (median [IQR]) profiles over time in the efficacy population. eGFR (**A**) in male and female patients, (**B**) stratified by baseline kidney function (eGFR > 120 mL/min/1.73 m^2^ vs. ≤ 120 mL/min/1.73 m^2^), and (**C**) according to ADA status at baseline. ^a^The assessments at Weeks 80 and 132 were added as a protocol amendment and implemented after most patients were past these study timepoints. ADA, anti-drug antibody; eGFR, estimated glomerular filtration rate; IQR, interquartile range

The median (IQR) change in eGFR from baseline (*N = *29) was ‒1.9 (‒5.4; 1.8, *n = *28) mL/min/1.73 m^2^ at Week 52 (Year 1) and ‒11.1 (‒15.1; ‒6.5, *n = *21) mL/min/1.73 m^2^ at Week 208 (Year 4). At most timepoints, absolute eGFR values were higher in males than females (Fig. 2A); the respective median (IQR) changes were ‒2.4 (‒4.5; 1.8, *n = *22) vs. ‒0.7 (‒6.3; 1.8, *n = *6) mL/min/1.73 m^2^ at Week 52 and ‒11.7 (‒15.1; ‒3.4, *n = *17) vs.‒10.9 (‒14.2; ‒10.3, *n = *4) mL/min/1.73 m^2^ at Week 208. All patients with eGFR values > 120 mL/min/1.73 m^2^ at baseline were male (*n* = 5; 4/5 ADA-positive) and had median (IQR) changes of ‒3.7 (‒7.2; ‒3.3, *n = *5) and ‒12.6 (‒14.1; ‒7.4, *n = *4) mL/min/1.73 m^2^ at Week 52 and Week 208, respectively, whereas patients with baseline eGFR ≤ 120 mL/min/1.73 m^2^ (*n = *24) showed changes of ‒0.7 (‒4.5; 2.5, *n = *23) and ‒10.8 (‒15.1; ‒6.5, *n = *17) mL/min/1.73 m^2^, respectively (Fig. 2B). A greater eGFR decline was observed in ADA-positive (all male, 4/9 with baseline eGFR > 120 mL/min/1.73 m^2^) vs. ADA-negative patients (Fig. 2C), with the respective median (IQR) changes of ‒3.2 (‒8.8; ‒2.4, *n = *8) vs. ‒0.7 (‒4.3; 2.2, *n = *20) mL/min/1.73 m^2^ at Week 52 and ‒12.6 (‒13.8; ‒3.4, *n = *7) vs. ‒10.9 (‒15.5; ‒6.5, *n = *14) mL/min/1.73 m^2^ at Week 208.

Shifts in eGFR categories and corresponding chronic kidney disease stages [40] from baseline to Weeks 52, 108, and 160 are presented in Table 3. Upward shifts were observed in patients with 30 < eGFR ≤ 60 and 60 < eGFR ≤ 90 mL/min/1.73 m^2^ and downward shifts in those with 90 < eGFR ≤ 120 and eGFR > 120 mL/min/1.73 m^2^. The patient whose dosing regimen was modified to pegunigalsidase alfa 1 mg/kg E2W due to deterioration in kidney function had the lowest eGFR values throughout the study, with 30.3, 23.8, and 4.8 mL/min/1.73 m^2^ at baseline, Week 52, and Week 208, respectively. The patient had a medical history of ongoing CKD, and their baseline UPCR value was 1.3 g/g. Following 92 weeks of study participation, the patient was diagnosed with end-stage renal disease and dialysis therapy was initiated.

**Table 3:** Shifts in eGFR status from baseline (efficacy population)

**eGFR,**^**a**^ **mL/min/1.73 m**^**2**^	eGFR status at baseline, *n* (%)			
Status at Week 52	30 < eGFR ≤ 60 CKD 3 *n = ***2**	60 < eGFR ≤ 90 CKD 2 *n = ***5**	90 < eGFR ≤ 120 CKD 1 *n = ***17**	eGFR > 120 *n = ***5**
30 < eGFR ≤ 60	0 (0.0)	0 (0.0)	0 (0.0)	0 (0.0)
60 < eGFR ≤ 90	1 (100.0)	3 (60.0)	4 (23.5)	0 (0.0)
90 < eGFR ≤ 120	0 (0.0)	2 (40.0)	13 (76.5)	2 (40.0)
eGFR > 120	0 (0.0)	0 (0.0)	0 (0.0)	3 (60.0)
**Status at Week 108**	**30 < eGFR ≤ 60** **CKD 3** ***n = * 1**	**60 < eGFR ≤ 90** **CKD 2** ***n = * 5**	**90 < eGFR ≤ 120** **CKD 1** ***n = * 17**	**eGFR > 120** ***n = * 3**
30 < eGFR ≤ 60	0 (0.0)	0 (0.0)	0 (0.0)	0 (0.0)
60 < eGFR ≤ 90	1 (100.0)	4 (80.0)	6 (35.3)	0 (0.0)
90 < eGFR ≤ 120	0 (0.0)	1 (20.0)	11 (64.7)	1 (33.3)
eGFR > 120	0 (0.0)	0 (0.0)	0 (0.0)	2 (66.7)
**Status at Week 160**	**30 < eGFR ≤ 60** **CKD 3** ***n = * 1**	**60 < eGFR ≤ 90** **CKD 2** ***n = * 4**	**90 < eGFR ≤ 120** **CKD 1** ***n = * 17**	**eGFR > 120** ***n = * 4**
30 < eGFR ≤ 60	0 (0.0)	0 (0.0)	0 (0.0)	0 (0.0)
60 < eGFR ≤ 90	1 (100.0)	4 (100.0)	5 (29.4)	0 (0.0)
90 < eGFR ≤ 120	0 (0.0)	0 (0.0)	12 (70.6)	3 (75.0)
eGFR > 120	0 (0.0)	0 (0.0)	0 (0.0)	1 (25.0)

eGFR calculated using the CKD-EPI equation; units are mL/min/1.73 m^2^

Abbreviations: CKD 1–3, chronic kidney disease, stage 1–3; CKD-EPI, Chronic Kidney Disease – Epidemiology Collaboration; eGFR, estimated glomerular filtration rate

#### Renal function: annualized eGFR slope

**Fig. 3:**
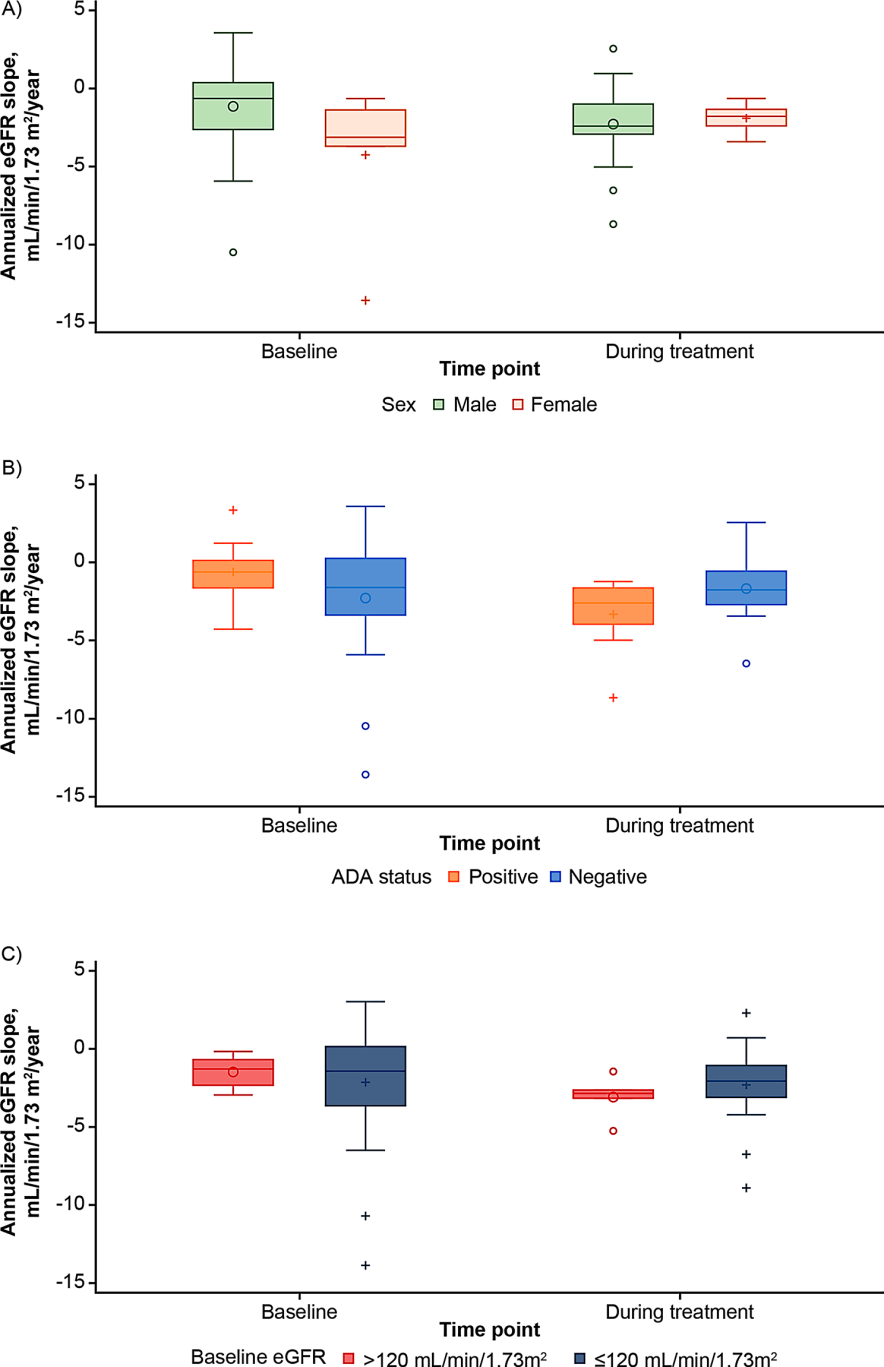
Annualized eGFR slope values in the efficacy population. eGFR slope stratified by **A**) sex^a^, **B**) ADA status^b^, and **C**) baseline kidney function (eGFR > 120 vs. ≤ 120 mL/min/1.73 m^2^)^c^. ^a^Male patients, *n = *23; female patients, *n = *6. ^b^ADA-positive patients, *n = *9; ADA-negative patients, *n = *20. ^c^Patients with baseline eGFR > 120 mL/min/1.73 m^2^, *n = *5; patients with baseline eGFR ≤ 120 mL/min/1.73 m^2^, *n = *24. *Note*: the baseline (pre-switch) annualized eGFR slope values were derived from inhomogeneous data sources (data obtained during the screening period and baseline visit from both central and local laboratories). ADA, anti-drug antibody; eGFR, estimated glomerular filtration rate

Overall, the median (IQR) annualized eGFR slope during treatment was ‒2.2 (‒2.9; ‒1.1) mL/min/1.73 m^2^/year. The values were ‒2.4 (‒2.9; ‒1.0) mL/min/1.73 m^2^/year in males (*n* = 23) and ‒1.8 (‒2.4; ‒1.3) mL/min/1.73 m^2^/year in females (*n* = 6; Fig. 3A). By ADA status, the values were ‒2.6 (‒4.0; ‒1.7) mL/min/1.73 m^2^/year in the ADA-positive subgroup (*n = *9, all males previously treated with agalsidase beta, 4 of whom had baseline eGFR > 120 mL/min/1.73 m^2^) and ‒1.8 (‒2.7; ‒0.6) mL/min/1.73 m^2^/year in the ADA-negative subgroup (*n = *20; Fig. 3B). In patients with baseline eGFR > 120 mL/min/1.73 m^2^ (*n = *5), the annual eGFR slope during treatment was ‒2.6 (‒2.9; ‒2.4) compared with ‒1.8 (‒2.8; ‒0.8) mL/min/1.73 m^2^/year in patients with baseline eGFR ≤ 120 mL/min/1.73 m^2^ (*n = *24, Fig. 3C).

#### Renal function: UPCR

Most patients had normal to mildly increased UPCR at baseline and throughout the study (Table 4). Shifts in UPCR categories from baseline to Weeks 52 and 208 are shown in Table 4.

**Table 4:** Shifts in the UPCR category (efficacy population)

**UPCR**^**a**^	UPCR category at baseline, *n* (%)		
Category at Week 52	Normal to mildly increased *n = ***24**	Moderately increased *n = ***3**	Severely increased *n = ***1**
Normal to mildly increased	18 (75.0)	1 (33.3)	0 (0.0)
Moderately increased	6 (25.0)	2 (66.7)	0 (0.0)
Severely increased	0 (0.0)	0 (0.0)	1 (100.0)
**Category at Week 208**	**Normal to mildly increased** ***n *= 18**^**b**^	**Moderately increased** ***n *= 3**	**Severely increased** ***n *= 1**
Normal to mildly increased	15 (83.3)	1 (33.3)	0 (0.0)
Moderately increased	2 (11.1)	2 (66.7)	1 (100.0)
Severely increased	1 (5.6)	0 (0.0)	0 (0.0)

^a^UPCR categorized according to KDIGO guidelines: normal to mildly increased < 0.15 g/g, 0.15 g/g ≤ moderately increased ≤ 0.5 g/g, and severely increased > 0.5 g/g

^b^All patients with data unavailable at Week 208 (*n = *6) had normal to mildly increased UPCR values at their last observation

Abbreviations: KDIGO, Kidney Disease: Improving Global Outcomes; UPCR, urine protein to creatinine ratio

#### Fabry disease biomarkers: plasma lyso-Gb3

**Fig. 4:**
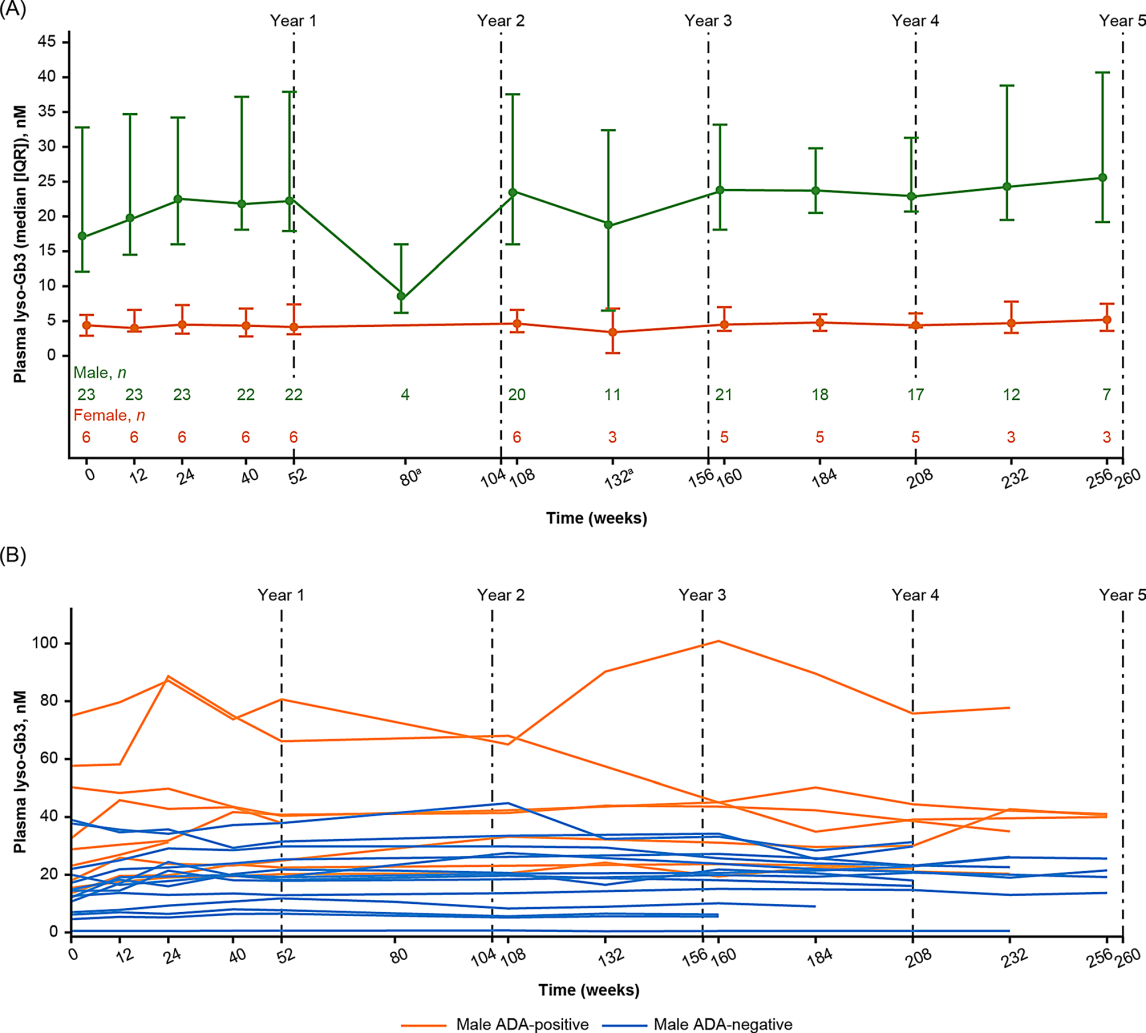
Plasma lyso-Gb3 concentrations over time in the efficacy population. Plasma lyso-Gb3 concentrations (**A**) stratified by sex (median [IQR]) and (**B**) in individual male patients according to ADA status at baseline. ^a^The assessments at Weeks 80 and 132 were added as a protocol amendment and implemented after most patients were past these study timepoints. ADA, anti-drug antibody; IQR, interquartile range; lyso-Gb3, globotriaosylsphingosine

In males, the median (IQR) change from baseline in plasma lyso-Gb3 concentration (normal range ≤ 2.4 nM [24]) was 5.1 (0.3; 7.8, *n = *22) nM at Week 52 and 3.2 (‒3.9; 8.5, *n = *17) nM at Week 208 (Fig. 4A), with greater variability observed in ADA-positive patients (Fig. 4B). The two male patients with the highest plasma lyso-Gb3 concentrations during the study had previously been treated with agalsidase beta and were ADA-positive at baseline and throughout the study. In females (all ADA-negative), plasma lyso-Gb3 concentrations were low and relatively stable (median [IQR] changes of ‒0.1 [‒0.4; 0.2, *n = *6] at Week 52 and ‒0.3 [‒1.0; 1.2, *n = *5] at Week 208; Fig. 4A).

#### Disease severity index and patient-reported outcomes

**Fig. 5:**
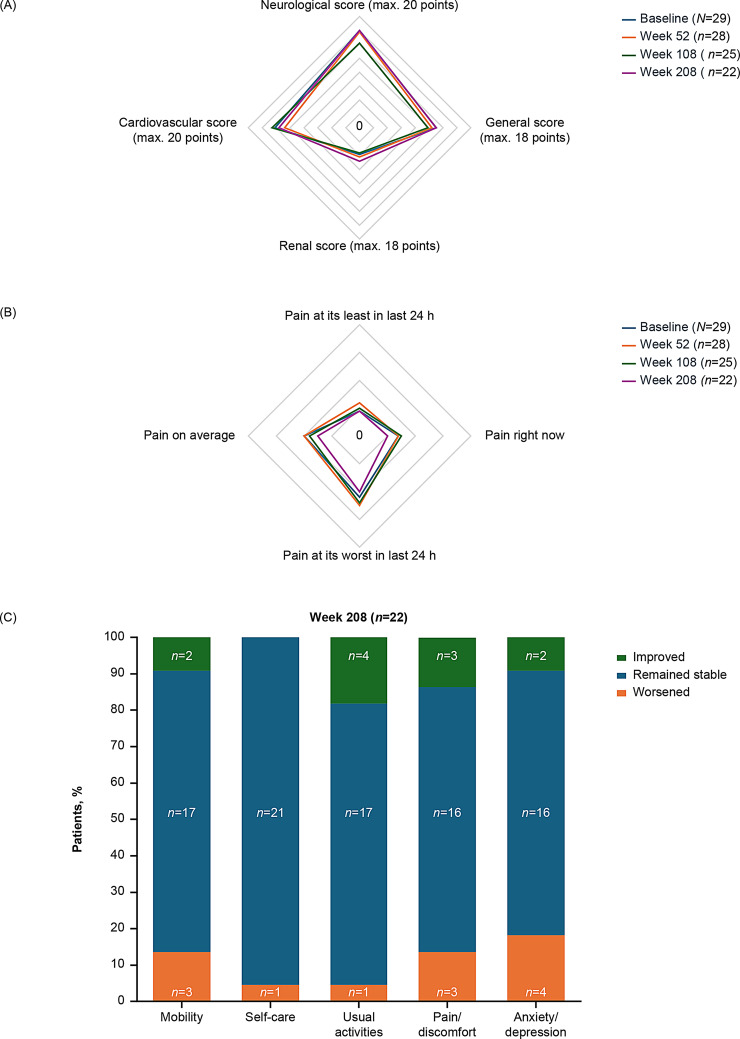
Disease severity and patient-reported outcomes in the efficacy population. (**A**) MSSI subdomain scores^a^ over the course of pegunigalsidase alfa treatment, (**B**) short form BPI severity domain scores^b^ over the course of pegunigalsidase alfa treatment, (**C**) EQ-5D-5L qualitative assessments (improvement, stability, or worsening) at Week 208 of pegunigalsidase alfa treatment. ^a^For all MSSI domains higher is worse. ^b^BPI severity scores ranged from 0 (no pain) to 10 (worst pain imaginable). MSSI, Mainz Symptom severity Index; BPI, Brief Pain Inventory; EQ-5D-5L, EuroQol 5-Dimensions 5-Levels Questionnaire; QoL, quality of life

Overall MSSI score remained stable over the study period, with a mean (SD) of 20.5 (9.7, *N = 29*) at baseline and a mean (SD) change from baseline to Week 208 of 0.6 (4.5, *n* = 22). Baseline MSSI scores were similar between ADA-positive (*n* = 9, all male and previously treated with agalsidase beta) and ADA-negative (*n* = 20) patients. By Week 208, ADA-positive patients showed a mean (SD) increase of 3.1 (3.6, *n = *7), whereas ADA-negative patients showed little change (‒0.5 [4.5, *n = *15]). Patients previously treated with agalsidase alfa (*n = *7) reported a decrease in MSSI by Week 208 (mean [SD] ‒3.3 [5.4], *n = *6), while those treated with agalsidase beta (*n* = 22) reported a modest increase (2.1 [3.2], *n = *16). Among patients who switched from agalsidase beta and were also ADA-negative at baseline, the mean (SD) MSSI score was 20.9 (7.6, *n =* 13) at baseline, with the mean (SD) change from baseline of 1.3 (2.7) at Week 208 (*n = *9). The mean MSSI subdomain scores for the overall population across the study are shown in Fig. 5A.

As assessed by the short form BPI, most patients reported improvement or no change in average pain relative to baseline, with 21/28 (75.0%) patients noting improvement or stability at Week 52 and 18/22 (81.8%) patients at Week 208. The mean (SD) average pain score was 2.0 (1.8, *N = *29) at baseline, with a mean (SD) change from baseline to Week 208 of ‒0.4 (1.5, *n* = 22). Slight improvements in pain scores over time were noted in both ADA-positive and ADA-negative patients, with the respective changes from baseline of ‒0.3 (1.6, *n = *7) and ‒0.4 (1.6, *n = *15) at Week 208. The mean BPI severity domain scores for the overall population across the study are shown in Fig. 5B, with small improvements recorded from Week 108 to Week 208.

For the BPI scores of the two patients whose regimen was modified to pegunigalsidase alfa 1 mg/kg E2W due to pain crisis episodes, the average pain scores ranged from 1.0–6.0 in one patient and from 0.0–1.0 in the other.

For the EQ-5D-5L QoL questionnaire, the mean (SD) overall health score was 78.3 (16.9, *N *= 29) at baseline, with a mean (SD) change from baseline of 4.1 (12.8, *n* = 22) at Week 208. The mean (SD) scores at baseline were 73.8 (24.2, *n = *9) in ADA-positive vs. 80.4 (12.5, *n = *20) in ADA-negative patients, with mean (SD) changes from baseline of 3.0 (15.8, *n* = 7) and 4.6 (11.7, *n* = 15) at Week 208, respectively. Improvement, stability, or worsening of the EQ-5D-5L score dimensions throughout the study in the overall efficacy population are presented in Fig. 5C.

### Safety

The median individual exposure to pegunigalsidase alfa in the safety population was 57.2 person-months, ranging from 35.9 to 60.8 person-months.

#### Adverse events

Overall, 477 TEAEs were reported in 28/29 (96.6%) patients (Table 5). The majority (467/477 [97.9%]) of TEAEs were assessed as mild or moderate in intensity and were resolved or resolving at the time of data cutoff.

**Table 5:** Summary of treatment-emergent adverse events (safety population)

	Patients, *n* (%) *N = ***29**	Events, *n* (rate per 100 patient-years)
At least 1 TEAE	28 (96.6)	477 (365.1)
At least 1 related TEAE^a^	13 (44.8)	51 (39.0)
At least 1 severe TEAE^b^	6 (20.7)	10 (7.7)
At least 1 severe, related TEAE^a,b^	0 (0.0)	0 (0.0)
At least 1 serious TEAE^c^	9 (31.0)	15 (11.5)
At least 1 serious, related TEAE^a,c^	0 (0.0)	0 (0.0)
At least 1 non-serious TEAE^c^	28 (96.6)	462 (353.6)
At least 1 non-serious, related TEAE^a,c^	13 (44.8)	51 (39.0)
At least 1 TEAE leading to study withdrawal	0 (0.0)	0 (0.0)
At least 1 TEAE leading to death	0 (0.0)	0 (0.0)

^a^Related TEAEs included events that were possibly, probably, or definitely related to study treatment. If causality was missing, the TEAE was considered related to the study drug. ^b^The “severe” category also included events classified as “very severe” (Grade 4) or fatal (Grade 5) according to CTCAE. If information on severity was missing, the TEAE was considered severe. ^c^If information on seriousness was missing, the TEAE was considered serious

Abbreviations: CTCAE, Common Terminology Criteria for Adverse Events; TEAE, treatment-emergent adverse event

A total of 10 severe TEAEs were reported in 6 (20.7%) patients, and 15 serious TEAEs (8 of which were also severe) occurred in 9 (31.0%) patients, who were all male (Table 6). None of the serious or severe TEAEs were treatment-related. Overall, 51 TEAEs in 13 (44.8%) patients were considered related to study treatment; all were mild or moderate in severity. No TEAEs resulted in study withdrawal or death.

**Table 6:** Summary of severe and serious TEAEs (safety population)

**System organ class**^**a**^ Preferred term	**Severe**^**b**^		Serious	
	Patients, *n* (%) *N = ***29**	Events, *n* (rate per 100 patient-years)	Patients, *n* (%) *N = ***29**	Events, *n* (rate per 100 patient-years)
**At least one TEAE**	**6 (20.7)**	**10 (7.7)**	**9 (31.0)**	**15 (11.5)**
**Cardiac disorders** Atrial fibrillation	**0** 0	**0** 0	**1 (3.4)** 1 (3.4)	**1 (0.8)** 1 (0.8)
**Congenital, familial, and genetic disorders** Fabry’s disease^c^	**1 (3.4)** 1 (3.4)	**1 (0.8)** 1 (0.8)	**1 (3.4)** 1 (3.4)	**1 (0.8)** 1 (0.8)
**Gastrointestinal disorders** Hypoesthesia oral Ileus	**0** 0 0	**0** 0 0	**2 (6.9)** 1 (3.4) 1 (3.4)	**2 (1.5)** 1 (0.8) 1 (0.8)
**General disorders and administration site conditions** Pyrexia	**1 (3.4)** 1 (3.4)	**1 (0.8)** 1 (0.8)	**0** 0	**0** 0
**Infections and infestations** Osteomyelitis Peritonitis bacterial Pharyngitis streptococcal Sepsis	**3 (10.3)** 0 1 (3.4) 1 (3.4) 1 (3.4)	**3 (2.3)** 0 1 (0.8) 1 (0.8) 1 (0.8)	**4 (13.8)** 1 (3.4) 1 (3.4) 1 (3.4) 1 (3.4)	**4 (3.1)** 1 (0.8) 1 (0.8) 1 (0.8) 1 (0.8)
**Injury, poisoning, and procedural complications** Infusion-related reaction^d^ Overdose	**1 (3.4)** 1 (3.4) 0	**1 (0.8)** 1 (0.8) 0	**1 (3.4)** 0 1 (3.4)	**1 (0.8)** 0 1 (0.8)
**Musculoskeletal and connective tissue disorders** Musculoskeletal chest pain	**0** 0	**0** 0	**1 (3.4)** 1 (3.4)	**1 (0.8)** 1 (0.8)
**Nervous system disorders** Cerebral infarction Cerebrovascular accident Epilepsy Seizure	**2 (6.9)** 1 (3.4) 0 1 (3.4) 1 (3.4)	**3 (2.3)** 1 (0.8) 0 1 (0.8) 1 (0.8)	**3 (10.3)** 1 (3.4) 1 (3.4) 1 (3.4) 1 (3.4)	**4 (3.1)** 1 (0.8) 1 (0.8) 1 (0.8) 1 (0.8)
**Renal and urinary disorders** End-stage renal disease	**1 (3.4)** 1 (3.4)	**1 (0.8)** 1 (0.8)	**1 (3.4)** 1 (3.4)	**1 (0.8)** 1 (0.8)

^a^Adverse events coded using the MedDRA dictionary (version 19.0). ^b^The severe category included events that were classified as severe, very severe, having life-threatening consequences, or fatal. ^c^Fabry’s disease: acute episodes of exacerbation of Fabry disease (e.g., fever pain crisis, Fabry pain episode). ^d^The infusion-related reaction manifested as generalized pain upon infusion initiation, but was adjudicated as unlikely to be related to study treatment by the investigator

Abbreviations: MedDRA, Medical Dictionary for Regulatory Activities; TEAE, treatment-emergent adverse event

#### Duration of infusion

Median (IQR) infusion duration decreased from 4.5 (4.5; 4.7) h at baseline to 2.0 (2.0; 2.1) h at Week 52, and remained steady throughout the OLE, with longer infusion times observed in patients with higher body weight (Table 7).

**Table 7:** Duration of infusion of pegunigalsidase alfa (2 mg/kg) according to body weight (safety population)

Infusion duration, h	≤ 100 kg **body weight**^**a**^	> 100 kg **body weight**^**a**^	Overall
**Baseline**			
* n*	24	5	29
Median (IQR)	4.5 (4.5; 4.6)	6.1 (6.1; 6.1)	4.5 (4.5; 4.7)
**Week 52**			
* n*	24	5	29
Median (IQR)	2.0 (2.0; 2.0)	3.0 (2.0; 3.2)	2.0 (2.0; 2.1)
**Week 208**			
* n*	19	6	25
Median (IQR)	2.1 (2.0; 2.3)	3.0 (2.0; 3.2)	2.1 (2.0; 2.3)

^a^For each infusion, the classification is based on the latest weight measurement available for the patient

Abbreviation: IQR, interquartile range

#### Infusion-related reactions

There were 43 infusion-related reactions reported in 9 (31.0%) patients (Table 8), all but one of whom were male. The rates were 2.7 vs. 0.9 infusion-related reactions per 100 infusions in male and female patients, respectively. Of the 9 patients with infusion-related reactions, 8 had previously been treated with agalsidase beta (2.9 infusion-related reactions per 100 infusions). Of those 8, 5 patients had pre-existing ADAs at baseline and experienced 37 out of the 43 infusion-related reactions (86.0%). Four of these 5 patients remained ADA-positive whilst receiving pegunigalsidase alfa treatment, whereas one seroreverted during the primary BRIGHT study. The remaining one patient, who had previously received agalsidase alfa, experienced a single infusion-related reaction (0.3 infusion-related reactions per 100 infusions) and was ADA-negative throughout the study. All infusion-related reactions were mild or moderate in severity, and all but one resolved as of the cutoff date for this interim analysis. The majority of infusion-related reactions (27 [62.8%]) occurred within the first year of treatment with pegunigalsidase alfa. The rate of infusion-related reactions in the primary BRIGHT study was 6.8 per 100 infusions (*N = *29), whereas during the OLE, the rates were generally low (1.8, 0.5, and 0.8 per 100 infusions during the 2nd [*N = *29], 3rd [*N = *29], and 4th year [*n = *28], respectively) and remained low after the 4th year of treatment (1.6 [*n = *25]).

**Table 8:** Summary of infusion-related reactions (safety population)

	Patients, *n* (%) *N = ***29**	Events, *n* (rate per 100 infusions)
At least 1 infusion-related reaction^a^	9 (31.0)	43 (2.4)
At least 1 mild or moderate infusion-related reaction	9 (31.0)	43 (2.4)
At least 1 severe infusion-related reaction^b^	0 (0.0)	0 (0.0)
At least 1 serious infusion-related reaction	0 (0.0)	0 (0.0)
At least 1 infusion-related reaction leading to study withdrawal	0 (0.0)	0 (0.0)
At least 1 infusion-related reaction leading to death	0 (0.0)	0 (0.0)

^a^Infusion-related reactions were defined as TEAEs occurring during the infusion or within 2 hours after the completion of the infusion that were reported as related to study treatment (excluding TEAEs defined as injection site reactions). ^b^The “severe” category also included events classified as “very severe” (Grade 4) or fatal (Grade 5) according to CTCAE

Abbreviations: CTCAE, Common Terminology Criteria for Adverse Events; TEAE, treatment-emergent adverse event

Most patients did not require premedication to manage infusion-related reactions. The proportion of patients who needed infusion premedication decreased from baseline (8/29 [27.6%] patients) to Week 52 (4/29 [13.8%] patients) and remained fairly stable at low levels thereafter (4/25 [16.0%] patients at Week 208).

#### Anti-drug antibodies

**Fig. 6:**
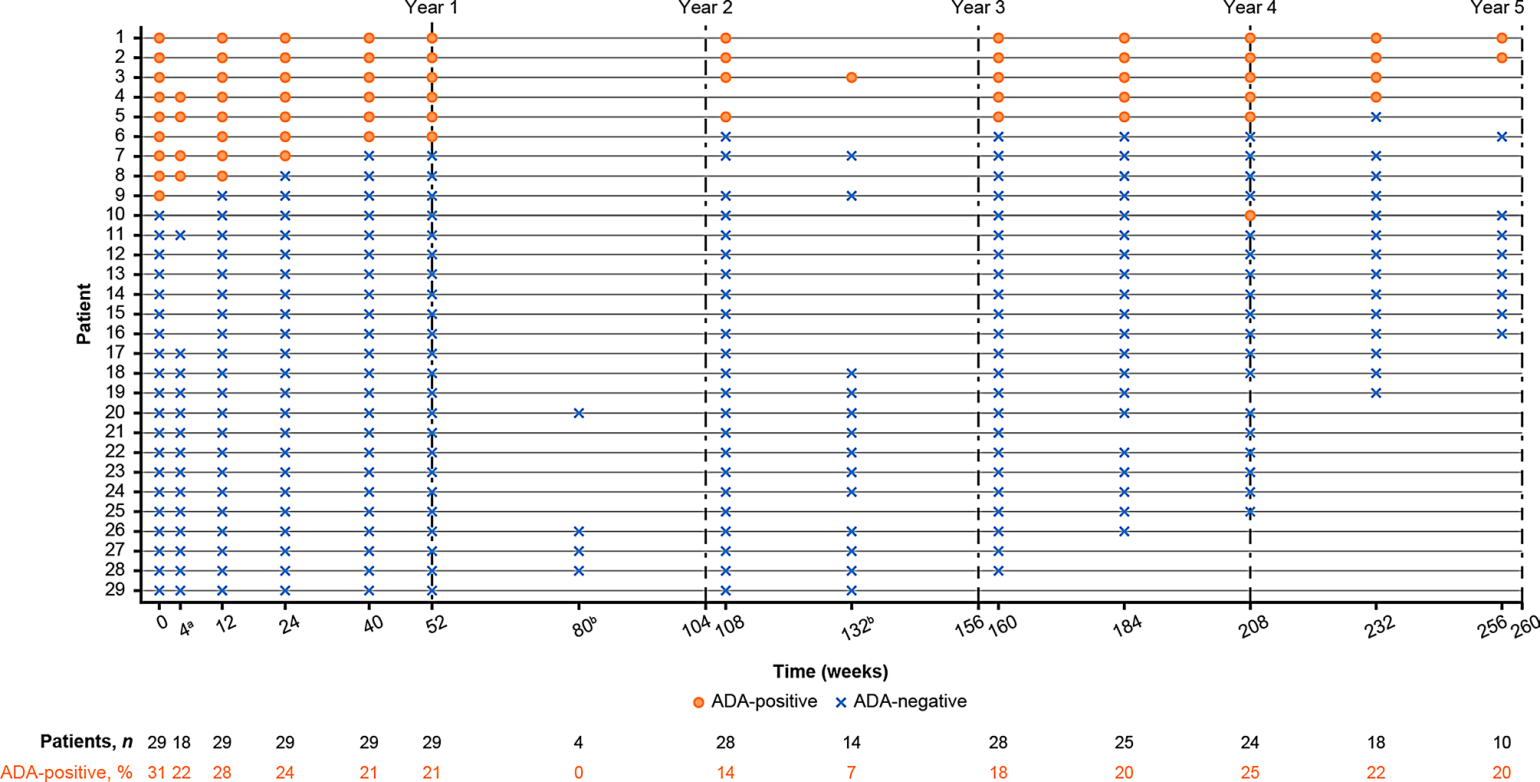
ADA status in individual patients and overall safety population during pegunigalsidase alfa treatment. ^a^The assessment at Week 4 was added as a protocol amendment of the primary BRIGHT study and implemented after some patients were past this study timepoint. ^b^The assessments at Weeks 80 and 132 were added as a protocol amendment of the OLE and implemented after most patients were past these study timepoints. Individual aassessments for some patients were missing due to the COVID-19 pandemic (for one patient) or unspecified reasons. ADA, anti-drug antibody; OLE, open-label extension

Figure 6 illustrates changes in ADA status over time in individual patients and the overall safety population. A total of 10 patients (34.5%) tested positive for ADAs at baseline; all were male and had previously received agalsidase beta. Nine of these patients were also positive for ADAs cross-reacting with pegunigalsidase alfa at baseline. All ADA-positive samples had antibodies targeting the α-Gal A enzyme backbone; one sample had antibodies to the PEG moieties of pegunigalsidase alfa. None of the samples were positive for antibodies against the plant glycans of the study drug. All but two ADA-positive samples had enzyme neutralizing activity at most timepoints of the study. Four of the 9 patients remained ADA-positive and 5 patients seroreverted. Of these 5 patients, 4 became ADA-negative during pegunigalsidase alfa treatment, and one patient was ADA-negative at all timepoints after baseline. One patient who was ADA-negative at baseline transiently developed treatment-induced non-neutralizing *de novo *ADAs at a single post-baseline timepoint (Week 208; titer: 266).

## Discussion

In the current OLE study, long-term treatment with pegunigalsidase alfa 2 mg/kg E4W for 3–5 years was well-tolerated and maintained disease stability in adult patients with Fabry disease at the interim analysis cutoff. These findings align with the evidence of BRIGHT [25] and, in turn, may support the feasibility of this alternative, less frequent administration schedule in patients with Fabry disease.

After 3–5 years of treatment, the change in eGFR and annualized eGFR slope as well as the UPCR results indicate that pegunigalsidase alfa 2 mg/kg E4W exerted a sustained treatment effect in stabilizing kidney function, in agreement with the kidney therapeutic goal derived from available natural history data on Fabry nephropathy and data from patients receiving approved ERTs [3, 11, 12, 41–44]. Annualized eGFR slope values during pegunigalsidase alfa treatment were more negative in certain subpopulations, i.e., males, ADA-positive patients, and those with baseline eGFR > 120 mL/min/1.73 m^2^. These findings align with literature showing a reduction of renal function in males and ADA-positive patients treated with ERTs E2W [41, 45]. However, average annualized eGFR slope values were less negative than ‒3 mL/min/1.73 m^2^/year in all subgroups, indicating a favorable treatment response [3, 44]. More negative annualized eGFR slope values in ADA-positive patients (all males) vs. ADA-negative patients denote a faster decline in kidney function, which may require more careful clinical monitoring.

Plasma lyso-Gb3 concentrations remained relatively stable over the study period. In line with previous studies [37–39], lyso-Gb3 levels were higher and more variable in males than females. Median plasma lyso-Gb3 concentrations showed a slight increase during the study, which was driven by values of two male patients who were ADA-positive throughout (Fig. 4B). Lyso-Gb3 levels have been found to correlate with disease severity, including cardiac and renal measures, and the metabolite is considered a reliable biomarker for monitoring Fabry disease progression [39]. Thus, it has been used in clinical studies to track and assess treatment response [23, 24, 46, 47]. However, further studies are required to establish clinically meaningful thresholds for monitoring treatment response, especially in ERT-experienced patients [39].

The overall MSSI scores showed stability over 3–5 years, with no indications of clinical deterioration. Mean changes from baseline were more likely to suggest improvements in agalsidase alfa vs. agalsidase beta pre-treated patients, and in ADA-negative vs. ADA-positive patients.

Improvement or stability in pain severity, as assessed by the short form BPI, were observed for most patients (~80%). These results are in line with long-term improvements in pain scores reported for other ERTs [6, 48] and pegunigalsidase alfa in ERT-naïve patients [20, 22]. However, the dosing regimen of pegunigalsidase alfa was modified to 1 mg/kg E2W in 2 patients due to several pain crises. For one of these patients, fewer TEAEs were reported after moving to the E2W dosing schedule, but no clear trends in changes in pain levels were observed. For the other patient, the insufficient duration of post-switch follow-up precludes reliable comparisons about pain between the dosing schedules. Generally, the low pain levels reported by the BPI suggest the occurrence of episodic (rather than chronic) pain in this patient. Besides pain crises, the dosing regimen was also modified for another patient due to deterioration of kidney function. This likely reflects further progression of their renal disease, as the patient had a medical history of ongoing CKD and proteinuria at study entry. Furthermore, the patient had a serious TEAE of end-stage renal disease after the dosing modification. Collectively, these findings highlight the importance of clinical condition monitoring of patients with Fabry disease during therapy.

No notable changes were observed in the EQ-5D-5L scores, indicating stable QoL. As a generic measure not specific to Fabry disease, EQ-5D-5L may not capture aspects of QoL that may change with less frequent administration regimen of pegunigalsidase alfa [48, 49]. Nonetheless, stable QoL supports the use of this regimen in maintaining disease stability.

The safety results were consistent with previous findings [20, 22–25] and showed that long-term treatment with pegunigalsidase alfa was well-tolerated, with no new safety signals. The reduced infusion duration achieved in the primary BRIGHT study [25] was maintained throughout the OLE, corroborating the continued tolerability of this dosing regimen. In line with data reported previously for pegunigalsidase alfa and agalsidase beta (both 1 mg/kg E2W), most infusion-related reactions occurred during the 1st year of treatment [22, 50], with low annual event rates recorded thereafter. Although the overall rate of infusion-related reactions in this study was higher than that reported previously in patients treated with the approved pegunigalsidase alfa dosing regimen of 1 mg/kg E2W (2.4 vs. 0.5 events per 100 infusions) [24], all recorded infusion-related reactions were mild or moderate in severity. During 3–5 years of treatment with pegunigalsidase alfa, 4/9 patients who had ADAs at baseline became ADA-negative. While switching to treatment with pegunigalsidase alfa E4W did not induce *de novo *ADA development in any patients participating in the primary BRIGHT study [25], one patient who was ADA-negative at baseline transiently developed treatment-induced, low-titer, non-neutralizing ADAs targeting the enzyme moiety of pegunigalsidase alfa at a single timepoint in the OLE. The reduction in the prevalence of ADAs, together with the low rate of long-term *de novo *ADA development, supports the favorable immunological profile of pegunigalsidase alfa [21, 22]. However, the use of diverse ADA assays limits cross-study comparisons.

This study is limited by the low number of patients, especially in the female cohort, as well as its open-label design and lack of a control group. Thus, the results of subgroup analyses should be evaluated with caution. Additionally, it should be noted that baseline (pre-switch) annualized eGFR slope values were derived from inhomogeneous data sources; historical and study data obtained during the screening period and baseline visit from both central and local laboratories were used.

## Conclusions

The interim results of this ongoing OLE study show that pegunigalsidase alfa 2 mg/kg administered E4W continues to be well-tolerated in stable adult patients with Fabry disease previously treated with other ERTs E2W, with no new safety signals reported. In most patients, disease signs and symptoms and QoL remained relatively stable during 3–5 years of treatment. The current results indicate that the 2 mg/kg E4W dosing regimen of pegunigalsidase alfa has a sustained treatment effect in stabilizing kidney function over time, especially in females and ADA-negative males, whereas additional prospective data are needed to better understand outcomes in ADA-positive males. Administration of pegunigalsidase alfa E4W should be accompanied by close clinical monitoring, especially in patients at high risk of progression. Any deterioration in clinical status should prompt clinical reassessment and, potentially, treatment modification to E2W infusions. The final results of this OLE study will provide further evidence on the long-term effectiveness of pegunigalsidase alfa 2 mg/kg E4W administration schedule.

## Electronic Supplementary Material

Below is the link to the electronic supplementary material.

Supplementary Material 1

## Data Availability

Any data requests received from external parties will be reviewed on a case-by-case basis. Chiesi reserves the right to deny requests for any and all legally appropriate reasons. Data requests that risk sharing participant level data or proprietary information will not be approved.
