# Supplementary material for: Long-term efficacy and safety of pegunigalsidase alfa administered every 4 weeks in adults with Fabry disease: results from up to 5 years of the BRIGHT F51 phase III, open-label extension study
Source: Orphanet J Rare Dis. 2026 Mar 20;21:185. doi: 10.1186/s13023-026-04303-8 (PMC13154429; doi:10.1186/s13023-026-04303-8)
Supplement: Supplementary file 1 — Supplementary Material 1 [file 13023_2026_4303_MOESM1_ESM.pdf]

Long-term efficacy and safety of pegunigalsidase alfa administered every 4 weeks in adults with Fabry disease: results from up to 5 years of the BRIGHT F51 phase III, open-label extension study

Supplemental information to Holida M et al. *Orphanet Journal of Rare Diseases* 2026.

This is a plain language summary of an article about the BRIGHT F51 study, which was published in a medical journal called *Orphanet Journal of Rare Diseases* in 2026.

How to say…

Pegunigalsidase alfa: "peh-GOO-nih-GAL-sud-ace AL-fuh"
Agalsidase alfa: "AY-gal-sud-ace AL-fuh"
Agalsidase beta: "AY-gal-sud-ace BAY-tuh"
Fabry: "FAB-ree"

What is Fabry disease?

Fabry disease is a rare genetic disorder caused by changes (known as mutations) in a gene called **galactosidase alpha (*GLA*)**. The *GLA* gene gives instructions to make an enzyme named **alpha-galactosidase A (α-Gal A)**. In people with Fabry disease, mutations in the *GLA* gene mean that the enzyme α-Gal A is either missing or stops working properly. The enzyme is unable to break down fats called **globotriaosylceramide** (Gb3) and **globotriaosylsphingosine** (lyso-Gb3). This leads to the build-up of these fats in cells and organs throughout the body. This can cause complications in the nervous system, heart, and kidneys that can become life-threatening. Many people with Fabry disease also experience ongoing symptoms such as tiredness, pain, and stomach or bowel problems.

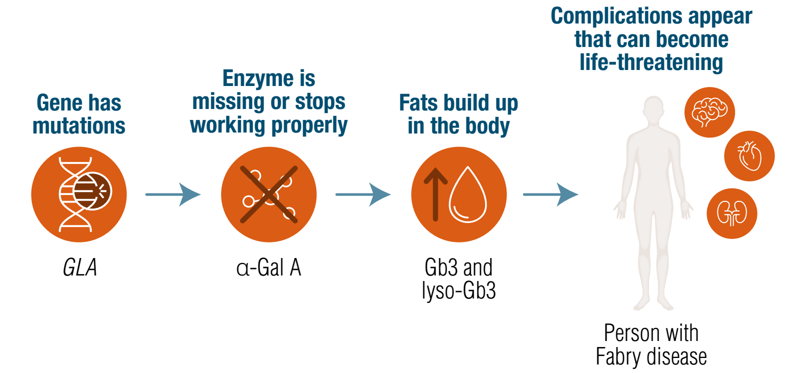

How is Fabry disease treated?

There are two types of treatment available for people with Fabry disease. One is an oral therapy, which only works in patients with specific *GLA* mutations, the other is **enzyme replacement therapy (ERT)**. ERT is delivered into the bloodstream – known as an **intravenous (IV) infusion** – where it provides a working version of the enzyme α-Gal A to the cells that are missing it. The enzyme can then break down the fats, Gb3 and lyso-Gb3, to prevent damage. There are three ERTs available to people with Fabry disease: agalsidase alfa, agalsidase beta, and pegunigalsidase alfa. They have shown to reduce complications and improve quality of life.

ERTs must be given through an IV every 2 weeks. Some people have reactions during or after the infusion. These reactions happen when the body responds negatively to the treatment. Such reactions can be mild, like fever or itching, but they can also be severe, like an allergic reaction that causes the body to go into shock. In some cases, the body's immune system recognizes the ERT as a foreign substance and makes **anti-drug antibodies (ADAs)**. ADAs can block the action of the ERT and reduce how well it works.

Pegunigalsidase alfa is different from the other ERTs because it is linked to a molecule called polyethylene glycol (PEG). This helps pegunigalsidase alfa stay in the blood longer, up to 80 hours, compared with 2 hours for the other ERTs. Because pegunigalsidase alfa lasts longer, researchers are investigating if it can be given to people with Fabry disease every 4 weeks rather than every 2 weeks.

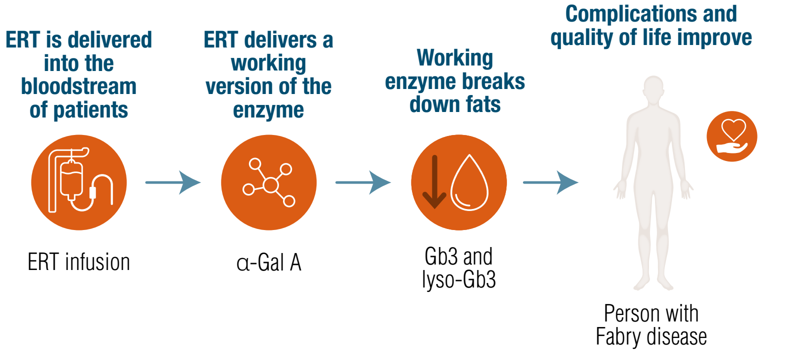

Why was the study done?

A previous study called BRIGHT looked at the effects of giving pegunigalsidase alfa at a dose of 2 mg per kg of body weight (2 mg/kg) every 4 weeks to participants with Fabry disease. These participants had been previously treated with agalsidase alfa or agalsidase beta every 2 weeks for at least 3 years before joining the study. The BRIGHT study was 1 year long and showed that switching participants from agalsidase alfa or agalsidase beta every 2 weeks to pegunigalsidase alfa every 4 weeks was well tolerated. It did not raise any new safety concerns and most participants' condition remained stable.

This study is an extension of the BRIGHT study. It is ongoing and follows the same participants to see how safe and effective pegunigalsidase alfa given every 4 weeks is in the long term. This analysis was done after 3 to 5 years of participation in the study.

What was the study plan?

Like BRIGHT, this was a **single-arm, open-label study**, meaning all participants were receiving the same treatment and both patients and researchers knew what treatment was being given.

If participants' condition worsened, they could switch from receiving pegunigalsidase alfa 2 mg/kg every 4 weeks to 1 mg/kg every 2 weeks, which is the dose currently approved by health authorities.

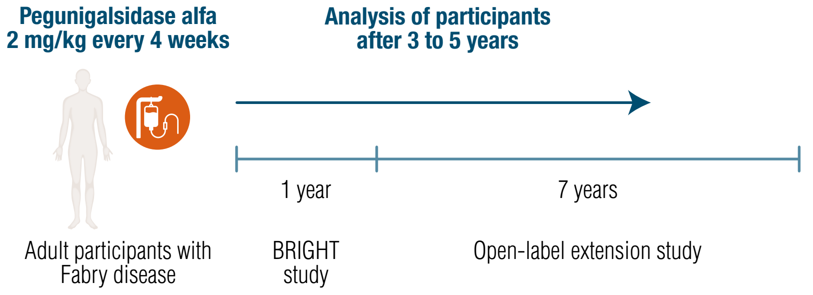

What did the researchers want to find out?

What happens when people with Fabry disease take pegunigalsidase alfa every 4 weeks for 3 to 5 years. In particular, how does this treatment affect:

- **kidney function?**
- **levels of lyso-Gb3 fat?**
- **quality of life?**
- **safety – were there any safety concerns with the treatment?**

Who were the participants in this analysis?

29 participants

Sex
23 males and 6 females

Age
19 to 58 years old, with an average age of 40.9 years

Previous therapy
7 participants had agalsidase alfa and 22 had agalsidase beta

ADAs at start of the study
9 participants had ADAs that react against pegunigalsidase alfa – these participants were males previously treated with agalsidase beta

This analysis included 29 participants who had completed the initial BRIGHT study. At the time of this analysis, 28 participants have received pegunigalsidase alfa treatment for at least 3 years.

What did the results show?

How did pegunigalsidase alfa affect participants' kidney function?

To look at participants' kidney function, the researchers used a measure called **estimated glomerular filtration rate (eGFR)**. eGFR shows how well the kidneys are filtering waste out of the body.

The eGFR slope shows how kidney function changes over time. If the slope goes down, this means kidney function is getting worse.

As people get older, their kidney function naturally gets worse, with eGFR decreasing by around 1 unit every year starting in their 40s. In people with Fabry disease, the decrease in eGFR is generally greater than 1 unit every year. This suggests their kidney function is getting worse faster than what is seen in healthy, aging people. Experts agree that kidney function can be considered stable in patients with Fabry disease if their eGFR slope does not decrease more than 3 units per year.

In this study, kidney function remained stable in most patients. The researchers found that kidney function got worse faster in some groups, such as men and people who had ADAs at the start of the study.

Kidney function in participants with Fabry disease treated with pegunigalsidase alfa

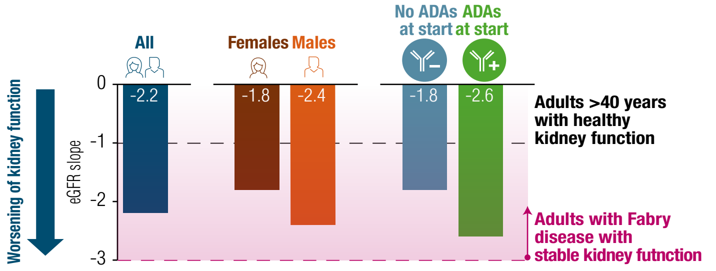

How did pegunigalsidase alfa affect the levels of lyso-Gb3 in participants?

Participants' levels of lyso-Gb3 stayed low and about the same during the study. Just like in earlier studies, lyso-Gb3 levels were higher and changed more over time in men than in women.

How did pegunigalsidase alfa affect participants' quality of life?

The researchers used questionnaires to ask participants how they felt about their pain and quality of life. Most participants (about 80%) said their pain improved or stayed the same. They also felt that their quality of life generally stayed the same.

What did this study tell us and why is it important?

The study showed that treatment with pegunigalsidase alfa at 2 mg/kg every 4 weeks for 3 to 5 years helped most people with Fabry disease keep their condition stable, with no new safety concerns. However, kidney function may get worse faster in some groups, like males and people who already have or develop ADAs. For this reason, patients' clinical condition should be carefully checked by doctors during treatment.

Administering pegunigalsidase alfa once every 4 weeks means that people with Fabry disease may not need to receive infusions as often as with other ERTs. Moving from treatment every 2 weeks to a once-a-month schedule could ease the treatment routine, save time and resources, and offer patients and their families greater flexibility. This less frequent dosing may also help improve quality of life by making treatment more convenient and easier to manage.

Did pegunigalsidase alfa treatment raise any safety concerns?

Any unintended medical effects that participants experience during a clinical study are called **adverse events**, even if a doctor decides they are not related to the study drug. If adverse events are life-threatening, cause long-term problems, or need hospital care, they are considered serious. Adverse events are also described as mild, moderate, or severe depending on how bad the symptoms are.

Most participants in this study (28 out of 29 participants) had at least one adverse event. However, only 13 participants had adverse events that were considered related to pegunigalsidase alfa. All of these related events (which included headache, pain in arms and legs, and nausea) were mild or moderate. No participants died during the study.

Infusion-related reactions are adverse events that happen during or within 2 hours of the infusion. These happened in 9 participants (8 men and 1 woman). All of these reactions were mild or moderate. Only one participant taking pegunigalsidase alfa developed new ADAs. These ADAs were only seen at one timepoint during the study.

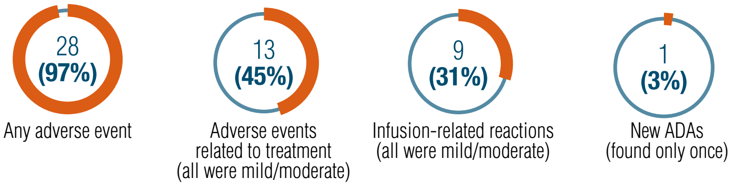

More information

This summary is based on the article called "Long-term efficacy and safety of pegunigalsidase alfa administered every 4 weeks in adults with Fabry disease: results from up to 5 years of the BRIGHT F51 phase III, open-label extension study" which was published in *Orphanet Journal of Rare Diseases* in 2026. To find out more about this extension study, please check https://clinicaltrials.gov/study/NCT03614234.

This study was sponsored by Protalix Biotherapeutics and Chiesi Farmaceutici. Medical writing support for the development of this summary, under the direction of the authors, was provided by Ashfield MedComms GmbH, an Inizio company, and funded by Chiesi. The authors thank all the study participants, their families, and the researchers. This summary was reviewed by the MPS Society and a member of the Fabry community.
The original authors of the full article reviewed and approved this summary.
